# Prediction of right recurrent laryngeal nerve lymph node metastasis in esophageal cancer based on computed tomography imaging histology

**DOI:** 10.3389/fonc.2024.1388355

**Published:** 2025-02-17

**Authors:** Xiaoli Huang, Shumin Jiang, Zhe Li, Xiong Lin, Zhipeng Chen, Chao Hu, Jianbing He, Chun Yan, Hongbing Duan, Sunkui Ke

**Affiliations:** ^1^ Department of Thoracic Surgery, Zhongshan Hospital of Xiamen University, Xiamen, Fujian, China; ^2^ The School of Clinical Medicine, Fujian Medical University, Fuzhou, Fujian, China

**Keywords:** esophageal cancer, right recurrent laryngeal nerve lymph nodes, computed tomography imaging histology, nomogram, Area Under Curve (AUC), CT

## Abstract

**Purpose:**

This study aimed to identify risk factors for right recurrent laryngeal nerve lymph node (RRLNLN) metastasis using computed tomography (CT) imaging histology and clinical data from patients with esophageal squamous cell carcinoma (ESCC), ultimately developing a clinical prediction model.

**Methods:**

Data were collected from 370 patients who underwent surgical resection at the Department of Thoracic Surgery, Zhongshan Hospital of Xiamen University, from December 2014 to December 2020. Subsequently, the venous-stage chest-enhanced CT images of the patients were imported into 3DSlicer 4.11 software, allowing for the extraction of imaging histological features. Additionally, by combining the clinical data of the patients, single- and multifactor analyses were conducted to screen the risk factors and build a predictive model in the form of a nomogram. The area under the curve (AUC) was used as a discriminant for model accuracy, while differentiation and calibration methods were applied to further evaluate the model’s accuracy. Finally, the Bootstrap resampling method was employed to repeat sampling 2,000 times to draw calibration curves, while the K-fold crossvalidation method was used for the internal validation of the prediction model.

**Results:**

The RRLNLN lymph node metastasis rate was 17.3%. Four significant factors—Maximum2DDiameterSlice, Mean, Imc1, and Dependence Entropy—were identified. Alignment diagrams were subsequently constructed, yielding an AUC of 0.938 and a C-index of 0.904 during internal validation.

**Conclusion:**

The model demonstrates high predictive accuracy, making it a valuable tool for guiding the development of preoperative protocols.

## 1 Introduction

Esophageal cancer is the sixth leading cause of cancer-related deaths and the eighth most common cancer worldwide. It affects more than 450,000 people all over the world, and its incidence is rising rapidly each year. The general 5-year survival rate for esophageal cancer ranges from 15% to 25% (1). According to some data, approximately half of the global cases of esophageal cancer occur in China, which has the highest number of patients in the world. Moreover, esophageal cancer ranks third in incidence rate and fourth in mortality rate among all cancers, with an extremely low 5-year survival rate. Hence, there is an urgent need for better treatment options (2). The existing treatment modality for esophageal cancer is mainly based on surgical resection with lymph node dissection and adjuvant radiotherapy. Presently, adopting esophageal cancer resection with three-field lymphadenectomy can improve the 5-year survival rate to approximately 40%–55.6%. However, this surgical approach is associated with an increased risk of complications (3–7). In particular, the clearance of lymph nodes close to the right recurrent laryngeal nerve (8–10) is prone to result in complications such as aspiration pneumonia, as indicated by related data (11). Moreover, according to some studies, not all lymph node dissections can bring postoperative survival benefits (9, 10). Therefore, it is of great importance to perform a preoperative prediction on two key items: whether the lymph nodes adjacent to the right recurrent laryngeal nerve are metastatic and whether this information can guide the development of a surgical plan, determine the surgical access, and refine the intraoperative lymph node dissection strategy. Currently, the most effective and conventional method for predicting metastasis of right recurrent laryngeal nerve lymph (RRLNLN) in esophageal cancer is chest computed tomography (CT), with the short-axis diameter redefined from 10 mm to 6.5 mm by Bin Li (12). However, in this regard, no consensus has been reached in domestic and international literature regarding this criterion, and CT shows low accuracy and specificity in predicting positive lymph nodes. The concept of imaging histology was first introduced by Dutch scholar Lambin (13) in 2012. It involves the use of imaging technologies such as CT, positron emission tomography (PET), nuclear magnetic resonance imaging (MRI), and B-ultrasound. Through these technologies, high-throughput image data are extracted, enabling tumor segmentation, feature extraction, and model building. This approach assists physicians in making the most accurate diagnosis by giving deeper mining, prediction, and analysis of the massive image data information. Considering the heterogeneity of tumors, imaging histology can be used in exploring the imaging data of patients, aiming at finding the internal characteristics of tumors and further reflecting the changes in the human microenvironment, cytological morphology, and gene level, thus affecting the diagnosis, treatment, and prognosis of diseases finally (14). At present, imaging features are extensively utilized in various fields of medical research, including the extended prediction of cerebral hemorrhage (15) and the development and verification of CT-based imaging features for predicting the overall survival of multiorgan tumors (16). This work aimed to predict the metastasis of RRLNLN and provide guidance for surgical planning by combining clinical data and imaging histological features of the patients.

## 2 Materials and methods

### 2.1 Materials

A total of 789 patients who underwent radical surgery for thoracic segmental esophageal cancer at the Department of Thoracic Surgery, Zhongshan Hospital of Xiamen University, from December 2014 to December 2020, were included in this retrospective analysis. After excluding those who did not meet the inclusion criteria, 367 patients were finally included in this study.

#### 2.1.1 Inclusion criteria

The inclusion criteria were as follows: (1) Patients were diagnosed with esophageal squamous carcinoma before surgery, and their data were complete and nondeficient, kept in the relevant electronic files or archived. (2) Patients accepted the relevant preoperative examinations, such as chest-enhanced CT, abdomen-enhanced CT, ECT, and neck ultrasound, to exclude distant metastases. (3) Patients underwent radical esophageal cancer surgery, including two- or three-field clearance, and pathological results indicated complete resection of the RRLNLN. (4) Patients received a chest CT enhancement scan at our hospital 3 weeks before surgery, and the image quality was clear enough to be exported in DICOM format.

#### 2.1.2 Exclusion criteria

The exclusion criteria were as follows: (1) Clinical examination data of the patients were missing, or the patients were examined outside the hospital. (2) Patients received preoperative neoadjuvant therapies, such as radiotherapy, chemotherapy, and immune-targeted therapy (pathological results of the patients changed after surgery, thus causing errors in the experimental results). (3) Patients suffered other malignant tumors in combination. (4) The RRLNLN was not identified in the patients, as confirmed by two pathologists and thoracic surgeons through analysis of the specimens obtained intraoperatively. (5) The esophageal cancer was located outside the thoracic segment, and the pathology was of a type other than squamous carcinoma.

### 2.2 Quantitative analysis of lymph node images

**Figure 1:**
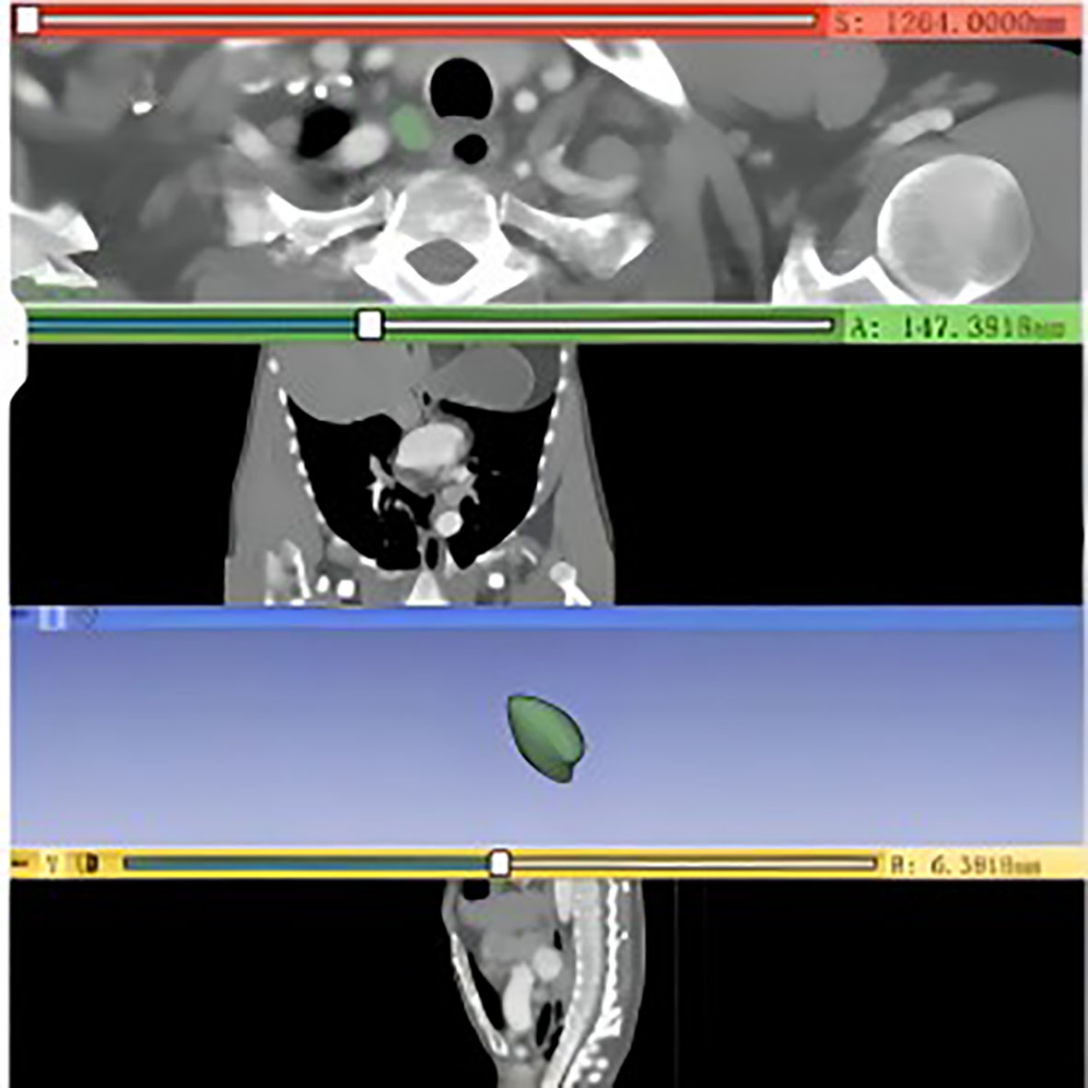
CT 3D reconstruction of the RRLNLN.

As shown in **Figure 1**, the high-quality and standardized CT images were first obtained in Digital Imaging and Communications in Medicine (DICOM) file format. Successively, these DICOM files were imported into 3DSlicer 4.11 software for automatic segmentation of the region of interest (ROI). Afterward, the lymph nodes were segmented into ROI regions by using a threshold segmentation method, followed by manual outlining of the ROI regions by two experienced thoracic surgeons. Finally, the outlined lymph nodes were given three-dimensional (3D) reconstruction, and high-throughput imaging features of the lymph nodes were automatically extracted and calculated using Radiomics. These features included first-order histogram features, morphological features, and parameters related to second- and high-order textural features, as well as the relationship between the lymph nodes and surrounding tissues. Based on the Radiomics analysis, a total of 130 imaging features were ultimately obtained.

### 2.3 Statistical analysis

Statistical analysis was conducted using R3.6.1 software. The count data were expressed as percentages, and the Chi-square test was performed for comparisons between groups. Normally distributed measures were presented as mean ± standard deviation, and two independent samples *t*-tests were used for comparisons between groups. The measures with skewed distribution were described by median and interquartile spacing, with the Mann–Whitney *U* test (Wilcoxon rank-sum test) used for comparisons between groups. Univariate analysis was performed on the demographic and clinical characteristics of the patients, and variables with *p* < 0.05 were included in the Lasso-logistic regression for variable screening, along with the imaging histological features extracted using Radiomics. Therefore, Lasso-logistic regression was performed by plotting the receiver operating characteristic curve (ROC) and calculating the area under the curve (AUC) to evaluate the model’s discrimination. The AUC value ranges from 0.5 to 1, with a value closer to 1 indicating better model discrimination and higher prediction accuracy. The predictors corresponding to the maximum AUC were chosen, and variables were screened using logistic regression and Lasso-logistic regression models, with predictors further filtered using the stepwise method. Additionally, internal validation of the model was performed using 10-fold crossvalidation. The R3.6.1 software rms package was used in building the alignment diagram prediction model. The Bootstrap method was used to repeat the sampling 2,000 times, generating the calibration curve for the internal validation of the alignment diagram model. Additionally, Harrell’s C-statistic was adopted to calculate the consistency index (C-index), assessing the discrimination of the alignment diagram model. All analyses were tested bilaterally, and statistical differences exhibited a two-sided *p* < 0.05.

## 3 Results

### 3.1 General characteristics of the cases

In this retrospective analysis, clinical study data from a total of 367 patients were obtained, as displayed in **Table 1**. Based on the metastasis of RRLNLN, patients were divided into two groups: metastatic (*n* = 67) and nonmetastatic (*n* = 300). As indicated by univariate analysis, five factors showed statistical significance (*p* < 0.05) between the two groups: the pathological type of hypofractionation, the longest diameter of lymph nodes, the lymph node plain CT value, the tumor plain CT value, and the tumor enhancement CT value. However, no statistically significant correlation was found between lymph node metastasis and other factors.

**Table 1:** Differences in demographic and clinical characteristics between the two groups.

Variables	Total	Metastatic group	Nonmetastatic group	*t*/*χ* ^2^	*p*-value
Gender					
Man	300 (81.74)	250 (82.51)	50 (78.13)	0.68	0.409
Woman	67 (18.26)	53 (17.49)	14 (21.88)		
Age (years)	61.28 ± 8.24	61.6 ± 7.97	59.73 ± 9.31	1.653	0.099
Smoking history					
Yes	258 (69.73)	217 (70.92)	41 (64.06)	1.178	0.278
None	112 (30.27)	89 (29.08)	23 (35.94)		
Tumor	3.8 ± 1.54	3.75 ± 1.59	4 ± 1.27	− 1.383	0.169
Longest diameter (cm)					
Tumor location	18 (4.86)	13 (4.25)	5 (7.81)	3.811	0.149
Upper	251 (67.84)	204 (66.67)	47 (73.44)		
Middle	101(27.30)	89(29.08)	12(18.75)		
Lower					
Degree of differentiation	9 (2.43)	8 (2.61)	1 (1.56)	–	0.001
Low	334 (90.27)	283 (92.48)	51 (79.69)		
Medium	27 (7.30)	15 (4.90)	12 (18.75)		
High	0.56 (0.5, 1)	0.55 (0.4, 0.7)	1.3 (1.1, 1.85)	11.027	< 0.001
Lymph node	35.35 ± 22.81	33.97 ± 23.09	42.06 ± 20.26	− 2.582	0.01
Longest diameter (cm)	62.84 ± 15.59	62.79 ± 15.43	63.1 ± 16.45	− 0.147	0.883
Lymph node plain CT value	19.9 ± 20.05	18.23 ± 20.74	27.85 ± 13.99	− 4.552	< 0.001
Lymph node enhancement CT value	39.12 ± 10.73	39.85 ± 10.77	35.66 ± 9.92	2.862	0.004

*p* < 0.05 indicates that the difference is statistically significant.

### 3.2 Lasso-logistic regression screening variables

**Figure 2:**
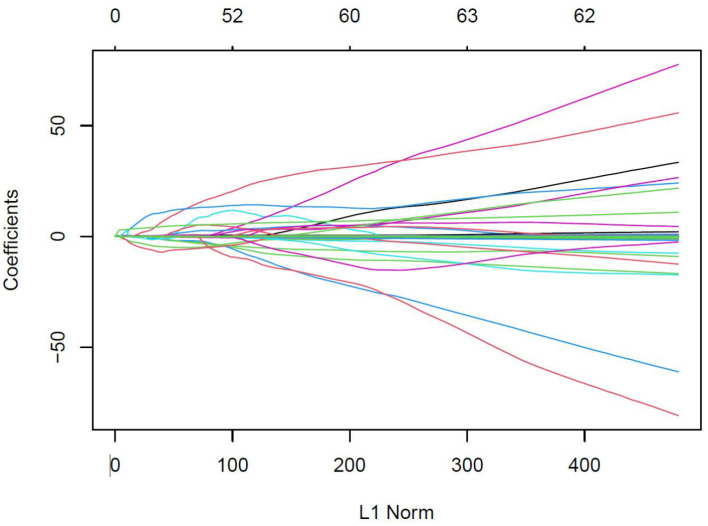
Eigencoefficient variation curves with log*λ* for the filtered features in the Lasso regression model. The vertical coordinate in the figure represents the respective values of the features in the model, the horizontal coordinate (L1 Norm) refers to the log Lambda, and the curve denotes the trajectory of each independent variable.

**Figure 3:**
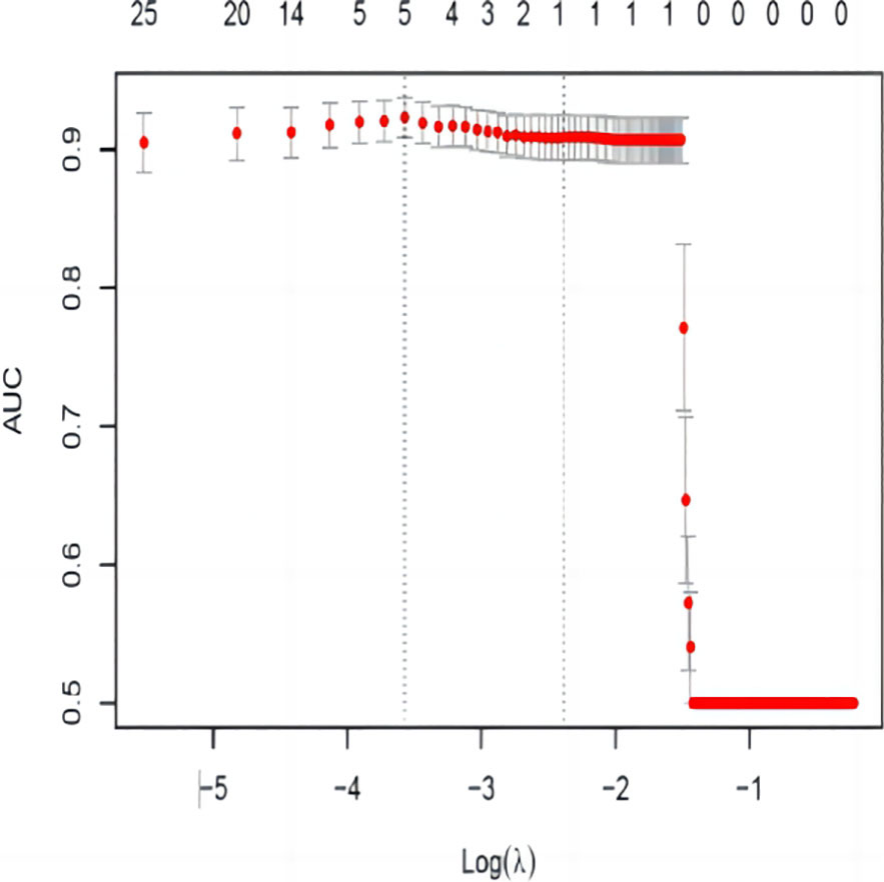
AUC variation curve with log*λ*. The dashed vertical line represents the minimum error *λ*, and the standard error *λ* corresponds to the optimal value.

As shown in **Figures 2**, **3**, variables with *p* < 0.05 from univariate analysis were included in Lasso-logistic regression for variable screening, alongside 130 imaging histological features. The Lasso-logistic regression analysis identified five variables with the maximum AUC value of 0.938: Mean, Maximum2DDiameterSlice, Imc1, Run Length Nonuniformity, and Dependence Entropy.

### 3.3 Results of logistic regression model analysis

The five variables (Mean, Maximum2DDiameterSlice, Imc1, Run Length Nonuniformity, and Dependence Entropy), which were screened using Lasso-logistic regression and the stepwise method, were included in the multifactor logistic regression analysis, with the results presented in **Table 2**. Based on the results from the multifactor logistic regression analysis, the prediction model was established with the following equation.

**Table 2:** Multivariate analysis of imaging parameters for predicting RRLNLN metastasis.

Variables	*β*	Standard error	Wald *χ* ^2^	*p*-value	OR (95% CI)
Constant	− 15.861	2.731	33.719	< 0.001	
Mean	− 0.004	0.001	13.542	< 0.001	0.996 (0.994,0.998)
Maximum 2D diameter slice	0.52	0.103	25.229	< 0.001	1.682 (1.373,2.060)
Imc1	0.079	0.034	5.338	0.021	1.083 (1.012,1.158)
Dependence entropy	1.554	0.429	13.145	< 0.001	4.733 (2.042,10.966)

*p* < 0.05 indicates that the difference is statistically significant.


$$p\left(\text{MPositive}\right)=\frac{e^{\left(-15.861-0.004\times \text{Mean}+0.520\times \text{Maximum}2\text{DDiameterSlice}+0.079\times \text{Imc}1+1.154\times \text{DependenceEntropy}\right)}}{1+e^{\left(-15.861-0.004\times \text{Mean}+0.520\times \text{Maximum}2\text{DDiameterSlice}+0.079\times \text{Imc}1+1.154\times \mathrm{DependenceEntropy}\right)}}$$

In our study, four imaging features (Mean, Maximum2DDiameterSlice, Imc1, Run Length Nonuniformity, and Dependence Entropy) were selected through the multivariate logistic regression model. These four predictors were then used to construct a nomogram model for predicting right recurrent laryngeal lymph node metastasis in esophageal cancer, enabling the prediction model to be transformed into a practical application tool. For example (**Figure 3**), the column diagram shows that the point (score) in the first row represents the score reference for each variable, and the total points represent the total score added by the scores corresponding to the values of all predictors. The predicted value represents the positive prediction probability, and the total score can be mapped to the linear predicted value based on the total points, thereby obtaining the probability of the patient’s right recurrent laryngeal lymph node metastasis. As shown in the figure below, a patient is considered positive when the total score is ≥ 220 (*p*(positive) ≥ 0.40).

### 3.4 Validation of model accuracy for predicting RRLNLN metastasis in esophageal cancer

**Figure 4:**
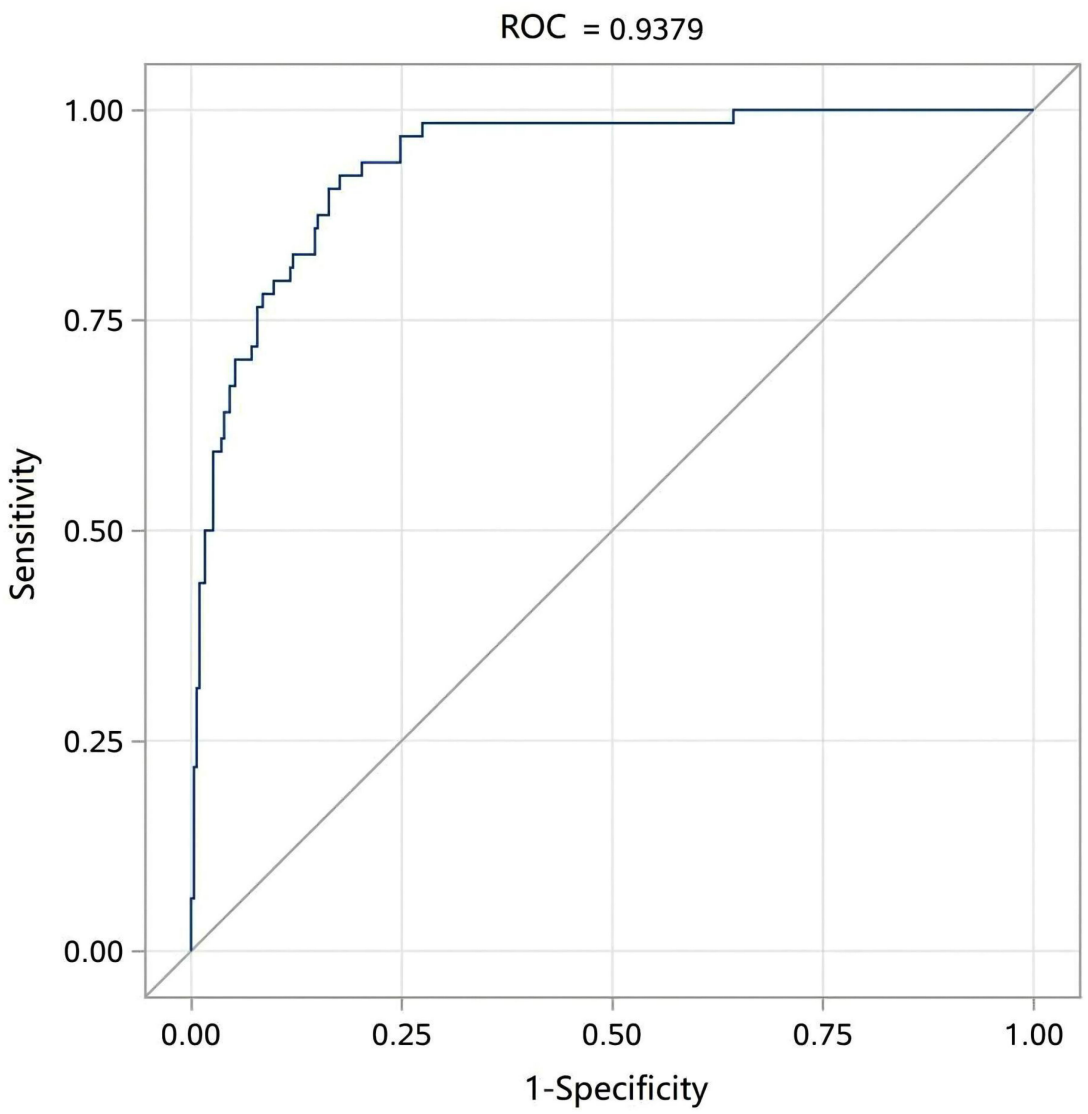
ROC curve analysis of the logistic regression model for evaluating model discrimination.

In this work, the ROC curve of the model was plotted, with an AUC value of 0.938 (95% CI = 0.909 to 0.967, *p* < 0.001), as shown in **Figure 4**. Therefore, the AUC value of 0.938, being greater than 0.9, indicates that the present prediction model has high accuracy and is considered positive when *p*(positive) ≥ 0.15 (i.e., the critical value). The Brier score of the model, calculated using R language, was 0.071, which is less than 0.25. The prediction results accorded well with the actual outcomes, indicating a high calibration degree of the model.

### 3.5 Internal validation of the model for predicting RRLNLN metastasis in esophageal cancer

In this work, the Bootstrap resampling method and K-fold crossvalidation were used for the internal validation of the prediction model.

**Figure 5:**
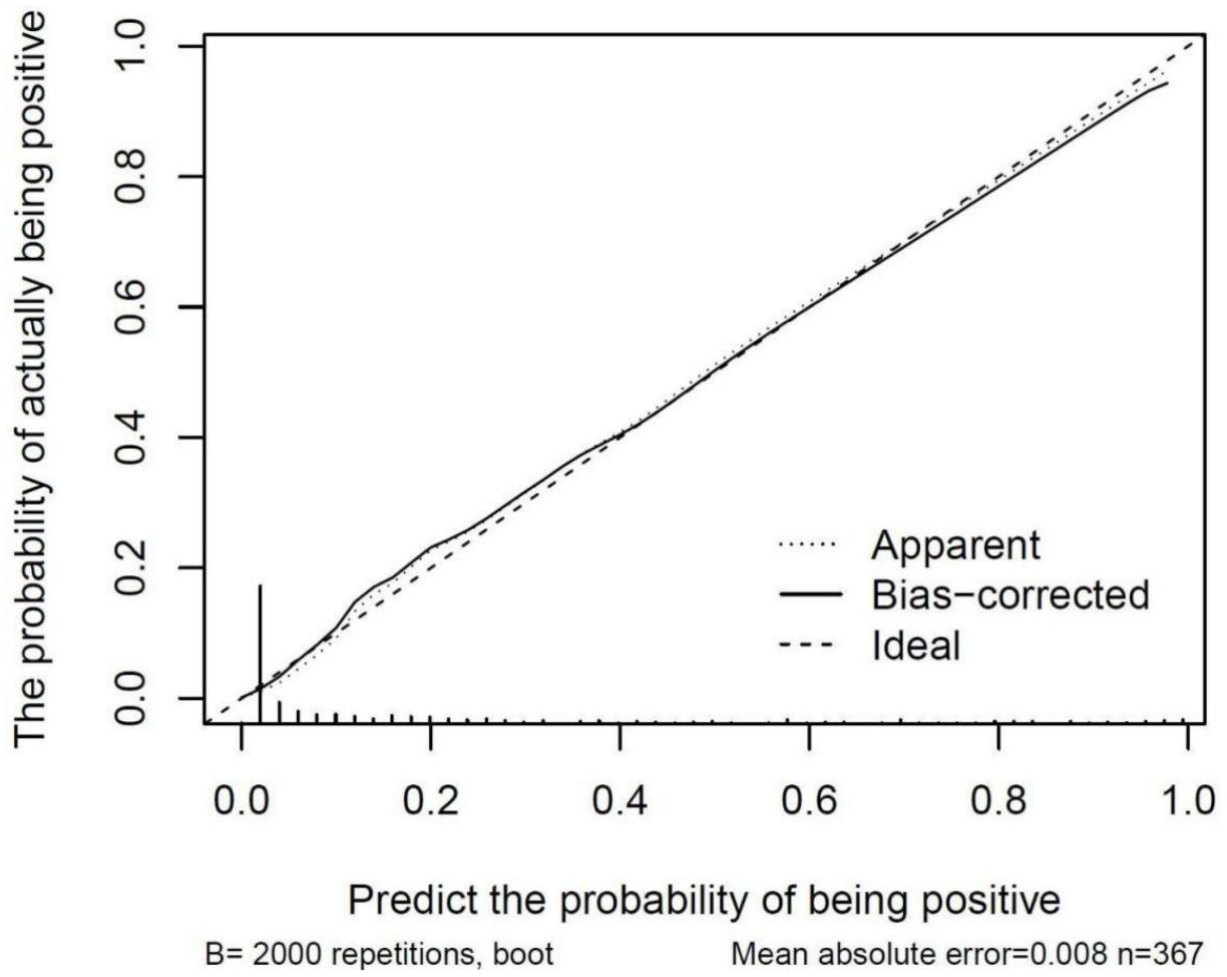
Calibration curve of the model for predicting RRLNLN metastasis in esophageal cancer.

As indicated in **Figure 5**, the dashed line represents the prediction result of the model, with the horizontal coordinate referring to the predicted positive probability and the vertical coordinate denoting the actual positive probability. Moreover, the dashed line (apparent) represents the result of internal validation, the 45°curve (ideal) refers to the perfect prediction of an ideal model, and the solid line (bias-corrected) indicates the bias-corrected result. As observed, the solid line and the dashed line overlapped almost completely, suggesting good internal validation of the model. The C-index of the prediction model was 0.904, with a mean absolute error of 0.008, indicating the high accuracy of the model in predicting RRLNLN metastasis in esophageal cancer.

In this work, the accuracy of the prediction model was evaluated using 10-fold crossvalidation, with the results presented in **Table 3**. According to the table, the accuracy of the model in the training set was 83.16%, the sensitivity was 91.65%, the specificity was 81.37%, the positive predictive value was 50.84%, and the negative predictive value was 97.90%. Moreover, the accuracy in the validation set was 81.64%, with a sensitivity of 91.55%. Additionally, the specificity was 80.67%, the positive predictive value was 50.71%, and the negative predictive value was 97.19%. As indicated, the results of the training and validation sets were similar, suggesting the high accuracy of the prediction model.

**Table 3:** Tenfold crossvalidation of the model for predicting RRLNLN metastasis.

	Accuracy (%)	Sensitivity (%)	Specificity (%)	Positive predictive value (%)	Negative predictive value (%)
Training set	83.16	91.65	81.37	50.84	97.90
Validation set	81.64	91.55	80.67	50.71	97.19

*p* < 0.05 indicates that the difference is statistically significant.

### 3.6 Establishment and use of a nomogram for predicting RRLNLN metastasis in esophageal cancer

**Figure 6:**
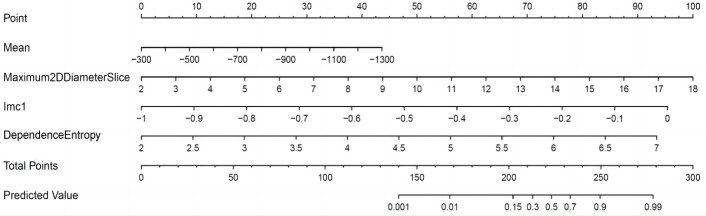
Nomogram for predicting RRLNLN metastasis in esophageal cancer.

As exhibited in the nomogram of **Figure 6**, the point in the first row represents the score reference for each variable, the total points refer to the total score derived from all the predictive factors, and the predicted value denotes the probability of a positive prediction. Therefore, the total score can be mapped to the linear score via the total points, thereby determining the probability of RRLNLN metastasis in the patient. According to the figure below, a patient was considered positive when the total score was not less than 202 (*p*(positive) ≥ 0.15).

## 4 Discussion

Esophageal lymphatic vessels form a lymphatic network in the lamina propria and submucosa, spreading across the intermuscular space (17, 18). The submucosal lymphatic network is present in the right tracheoesophageal groove and has a direct connection with the RRLNLN, as indicated (17, 19). Lymph nodes near the RRLN are more prone to metastasis compared with other groups of lymph nodes. Considering the special structure of the right recurrent laryngeal nerve, complications such as hoarseness, respiratory issues, anastomotic fistula, and atrial fibrillation can arise during its clearance. Therefore, preoperative determination of RRLNLN metastasis is crucial in the survival and prognosis of the patients.

Given the deep location of the RRLNLN, imaging modalities such as CT, MR, ultrasound, PET-CT, and EUS are the most conventional methods for preoperative metastasis determination. However, numerous studies indicate that PET-CT/CT provides a low reference value for RRLNLN metastasis (20–22). In recent years, with the increasing application of prediction models in clinical practice, many experts and scholars have developed models for predicting lymph node metastasis based on CT imaging features. Jing (23) draw a metastasis prediction model by combining imaging features, identifying four types of imaging parameters associated with lymph node metastasis: shape features, size features, intensity histogram, and textural features. Larger lymph node volumes are more likely to be invaded by tumors, as demonstrated in previous studies (24, 25). Among the textural features, one primarily indicates the complexity the tumor’s internal structure, which is closely associated with tumor activity and prognosis (26). Using this method, a clinical prediction model is innovatively constructed by extracting imaging features to predict lymph node metastasis. However, the AUC value of this model is only 0.79, suggesting low accuracy. Moreover, the model includes 11 predictors despite having only a few cases, making it challenging to apply clinically.

In this work, a new imaging histology model was developed using high-resolution enhanced CT images from our patients. Moreover, 3D Slicer 4.11 software was used to outline the RRLNLN in the original DICOM format of the CT images, realizing 3D reconstruction. Applying the Radiomics plug-in, the software automatically calculated the lymph node morphology, the textural features, and other relevant parameters, thereby facilitating the prediction of metastasis probability in the RLNLN. To address variable selection, the model complexity was adjusted to avoid overfitting and underfitting. Hence, Lasso-logistic regression was employed for multifactor analysis based on single-factor analysis, followed by a stepwise screening method to identify predictors corresponding to the maximum AUC. This approach maximized the scientific validity and accuracy of the screening process, ultimately leading to the construction of the alignment diagram for the prediction model. Simultaneously, the accuracy and consistency of the model were evaluated in the experiment. The results of the internal validation indicated that the alignment diagram could be widely used for preoperatively predicting RRLNLN metastasis of the right recurrent laryngeal nerve. Compared to previous methods, this approach was more intuitive and accurate. An AUC higher than 0.90 is generally considered highly accurate, and the AUC of this prediction model reached 0.938, indicating high accuracy. Moreover, according to relevant data, an internal validation C-index of 0.9 is also deemed highly accurate, and this model achieved a C-index of 0.904.

In this work, statistical analysis was conducted on a series of clinical data included in our univariate analysis, identifying five statistically significant factors (pathological type of hypofractionation): the longest diameter of lymph nodes, the lymph node plain CT value, the tumor plain CT value, the tumor enhancement CT value, and the longest diameter of lymph nodes. These findings are consistent with the results obtained in a previous study (27). Moreover, according to the statistical analysis, a strong correlation was found between the probability of lymph node metastasis and the pathological type of tumor, especially when the tumor was hypodifferentiated, as reported in previous studies (28, 29). The degree of tumor differentiation is more likely an independent risk element for thoracic lymph node metastasis in esophageal cancer, as it is negatively correlated with the rate of lymph node metastasis. A lower degree of differentiation indicates a higher degree of malignancy and a higher rate of lymph node metastasis. In this work, the CT values of tumor and lymph nodes were innovatively incorporated, including scan and enhancement CT values. Moreover, to explore the accuracy of the results, three target areas with the same scan and enhancement images were selected. The CT values of these areas were measured and averaged to obtain the final results. The CT value reflects the density of the object, and changes in density can be used to analyze the composition of the tumor. Generally, tumors are primarily composed of soft tissue, with CT values ranging from 30 to 100. Thus, the CT values of the tumor and lymph nodes were considered factors in this work. Statistical analysis indicated a close relationship between the CT values and RRLNLN metastasis.

In contrast to evaluation based solely on manual reading, the analysis of RRLNLN using imaging histological features was more comprehensive and refined. Moreover, by reconstructing the structure of the lymph nodes, the morphological and textural features were more adequately captured. Finally, a total of four imaging features were derived: the maximum two-dimensional (2D) diameter (slice) in the shape feature, the mean value in the first-order feature, the grayscale dependence entropy in the correlation matrix (GLCM) feature, and the correlation information measure 1 in the grayscale covariance matrix feature. These were identified as predictors for RRLNLN metastasis. In Radiomics, each of the four features represents the internal structure of the tumor. A larger value of the maximum 2D diameter (slice) in the shape feature represents more information contained in the image space. Therefore, uniform pixels arranged around a lymph node with a larger diameter may be associated with tumor metastasis components. First-order features, also known as voxel intensity features, are related to the gray-level frequency distribution within the painted target area. The mean value is usually used to describe the distribution of voxels in the target area, revealing the solid component of the lymph nodes. The GLCM is commonly employed to describe the two-by-two arrangement of voxels, with dependence entropy serving as a measure of the dependency on pro-domain intensity values. It also assesses the uniformity of image texture and the degree of randomness. It has been indicated that highly aggressive tumors with inhomogeneous internal echogenicity and enhanced entropy features in the texture show a stronger correlation with molecular typing (30). As a common method for describing texture by exploring the spatial correlation properties of the grayscale, the grayscale covariance matrix can reveal the content of real components contained within the tumor, specifically the number of tumor components present.

There are still limitations in this work. First, it is based on a single-center retrospective study with a relatively small sample size. Therefore, further prospective studies and multicenter studies with larger sample sizes are needed for training and validation. Second, the RRLNLN is small on the enhanced CT images, and some surrounding fat could not be eliminated even after correction by experienced technologists and clinicians. Therefore, further development of medical imaging technology and improvements in the accuracy and precision of tumor segmentation are required. Moreover, due to the lack of a larger public database, this model has only been internally validated, and further external validation is still pending.

## 5 Conclusion

3D Slicer 4.11 software was used to outline the ROI of lymph nodes, and imaging features were extracted from the ROI, including shape features, texture pattern heterogeneity, and intensity distribution. Moreover, a statistical method was employed to identify predictors for RRLNLN metastasis, and an alignment graph model was successfully constructed to predict RRLNLN metastasis preoperatively, thereby guiding the surgeon’s surgical plan and the patient’s prognosis for survival.

## Data Availability

The raw data supporting the conclusions of this article will be made available by the authors, without undue reservation.
